# Association of osteoporosis and varus inclination of the tibial plateau in postmenopausal women with advanced osteoarthritis of the knee

**DOI:** 10.1186/s12891-021-04090-2

**Published:** 2021-02-25

**Authors:** Shun-Ping Wang, Po-Kuan Wu, Cheng-Hung Lee, Cheng-Min Shih, Yung-Cheng Chiu, Cheng-En Hsu

**Affiliations:** 1 Department of Orthopedics, Taichung Veterans General Hospital, Taichung, Taiwan; 2 Sports Recreation and Health Management Continuing Studies-Bachelor’s Degree Completion Program, Tunghai University, Taichung, Taiwan; 3 Taichung Veterans General Hospital, Taichung, Taiwan; 4 Department of Food Science and Technology, Hung Kuang University, Taichung, Taiwan; 5 College of Biological Science and Technology, National Yang Ming Chiao Tung University, Hsinchu, Taiwan; 6 Department of Physical therapy, Hung Kuang University, Taichung, Taiwan; 7 School of Medicine, China Medical University, Taichung, Taiwan; 8 Department of Orthopedic Surgery, China Medical University Hospital, Taichung, Taiwan

**Keywords:** Osteoporosis, Osteoarthritis of knee, Arthroplasty, Lower extremity varus malalignment, varus inclination of the tibial plateau

## Abstract

**Background:**

Although varus inclination of the tibial plateau has increasingly been recognized as a major risk factor in the progression of Osteoarthritis of the knee (OA knee), little attention has been placed on the development of the varus inclination of the tibial plateau. Osteoporosis is a disease characterized by low bone mass and may increase the risk of a stress fracture in the proximal tibia. To date, risk factors for varus inclination of the tibial plateau are rarely reported. In this study, we investigated Bone Mineral Density (BMD) as a risk factor of varus inclination of the tibial plateau in postmenopausal women with advanced OA knee.

**Methods:**

A total of 90 postmenopausal women with varus OA knee who had received a total knee arthroplasty in our department between January 2016 and December 2019 were reviewed. Certain factors may correlate to inclination of the tibial plateau (Medial Tibial Plateau Angle, MTPA), including age, operation side, Kellgren-Lawrence grade of OA knee, BMD, Body Mass Index (BMI), Lateral Distal Femur Angle (LDFA), lower extremity alignment (Hip-Knee-Ankle angle, HKAA), and history of both spinal compression fracture and hip fracture were collected and analyzed.

**Results:**

Osteoporosis, lower extremity varus malalignment and age were significantly associated with varus inclination of the tibial plateau (MTPA) (*P* = 0.15, 0.013 and 0.033 respectively). For patients with a lower extremity varus malalignment (HKAA < 175°), osteoporosis (T-score ≤ -2.5) was significantly associated with inclination of the tibial plateau. For patients with a normal lower extremity alignment (HKAA ≥ 175°), no significant association was found between osteoporosis (T-score ≤ -2.5) and varus inclination of the tibial plateau.

**Conclusions:**

Osteoporosis, lower extremity varus malalignment and age are major risk factors for inclination of the tibial plateau in postmenopausal women with OA knee. More attention needs to be given to the progression of varus OA knee in postmenopausal women who simultaneously has osteoporosis and lower extremity varus malalignment.

## Background

Varus inclination of the tibial plateau is a common finding in patients with varus Osteoarthritis of the knee (OA knee) [1]. It is also recognized as a major risk factor for the onset and progression of osteoarthritis in patellofemoral joint and medial femoral-tibial joint [2–6]. Varus inclination of the tibial plateau is characterized by a remarkably decreased Medial Tibial Plateau Angle (MTPA). When the MTPA is less than 85°, the inclination of the tibial plateau becomes clinically significant and considered as a critical factor for the progression of OA knee [3, 6–8]. Early detection and intervention for inclination of the tibial plateau is crucial in preventing progression of osteoarthritis. However, risk factors for inclination of the tibial plateau are rarely reported in the available literature.

Aside from congenital and trauma origin, the main cause of varus inclination of the tibial plateau is insufficiency fracture [9–12]. Insufficiency fractures are the result of abnormal, cyclical loading on normal bone, which leads to local cortical resorption and fracture [13]. These fractures can cause severe pain, but can also be asymptomatic. [14, 15]. In the elderly population, insufficiency fractures are usually seen to be secondary to osteoporosis.

Osteoporosis is defined as low bone mass and the micro-architectural deterioration of bone tissue, with a consequent increase in bone fragility and susceptibility to fracture [16]. As well as OA knee, the prevalence of osteoporosis rises with age and menopausal status in women [17]. Osteoporosis is diagnosed radiographically by one’s Bone Mineral Density (BMD) T-score being ≤-2.5 clinically, which is associated with an elevated fracture risk [18]. Osteoporosis is generally known to be the main risk factor for low-energy trauma fractures, including fracture of the distal radius, vertebral body, proximal humerus and proximal femur [18].

To date, very few studies have reported risk factors for varus inclination of the tibial plateau in advanced OA knee patients. BMD is a popular and precise tool for the evaluation of osteoporosis. In this study, we investigated the association of BMD and varus inclination of the tibial plateau in postmenopausal women with advanced OA knee.

## Methods

### Patient enrollment

The medical records of patients with varus OA knee who had received a total knee arthroplasty in our hospital between January 2016 and September 2019 were reviewed retrospectively. The including criteria were postmenopausal women with a standardized whole leg weight bearing radiograph and Dual-energy X-ray Absorptiometry (DXA) [1] measurement for Bone Mineral Density (BMD). The exclusion criteria were obvious malalignment of the affected lower limb due to previous trauma and congenital diseases. Ultimately, a total of 90 patients were enrolled in our study. Certain factors may correlate to inclination of the tibial plateau (i.e. Medial Tibial Plateau Angle, MTPA) including age, operation side, Kellgren-Lawrence grade of OA knee, BMD, Body Mass Index (BMI), Lateral Distal Femur Angle (LDFA), Hip- Knee-Ankle Angle (HKAA), as well as history of both spinal compression fracture and hip fracture; the data of which were collected and analyzed.

### Measurements

**Fig. 1:**
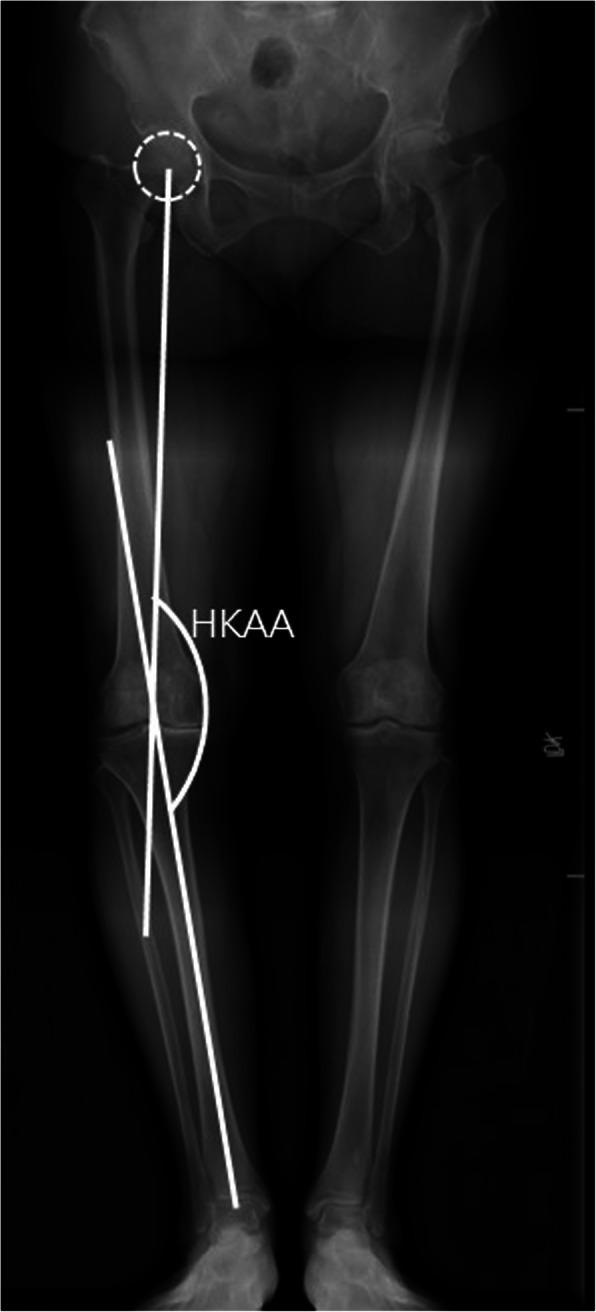
The hip-knee-ankle angle (HKAA) is measured on the whole-leg weight-bearing radiograph. It is defined as the medial angle between the mechanical axis line of the femur (direct line from the center of femoral head to the top of the femoral notch) and the mechanical axis line of the tibia (line from the ankle center to the center of the tibial spines)

The HKAA was defined as the medial angle between the mechanical axis line of the femur (direct line from the center of the femoral head to the top of the femoral notch) and the mechanical axis line of the tibia, i.e. line from the ankle center (mid-point of the talus in the coronal plane) to the center of the tibial spines [19]. (Fig. 1).

**Fig. 2:**
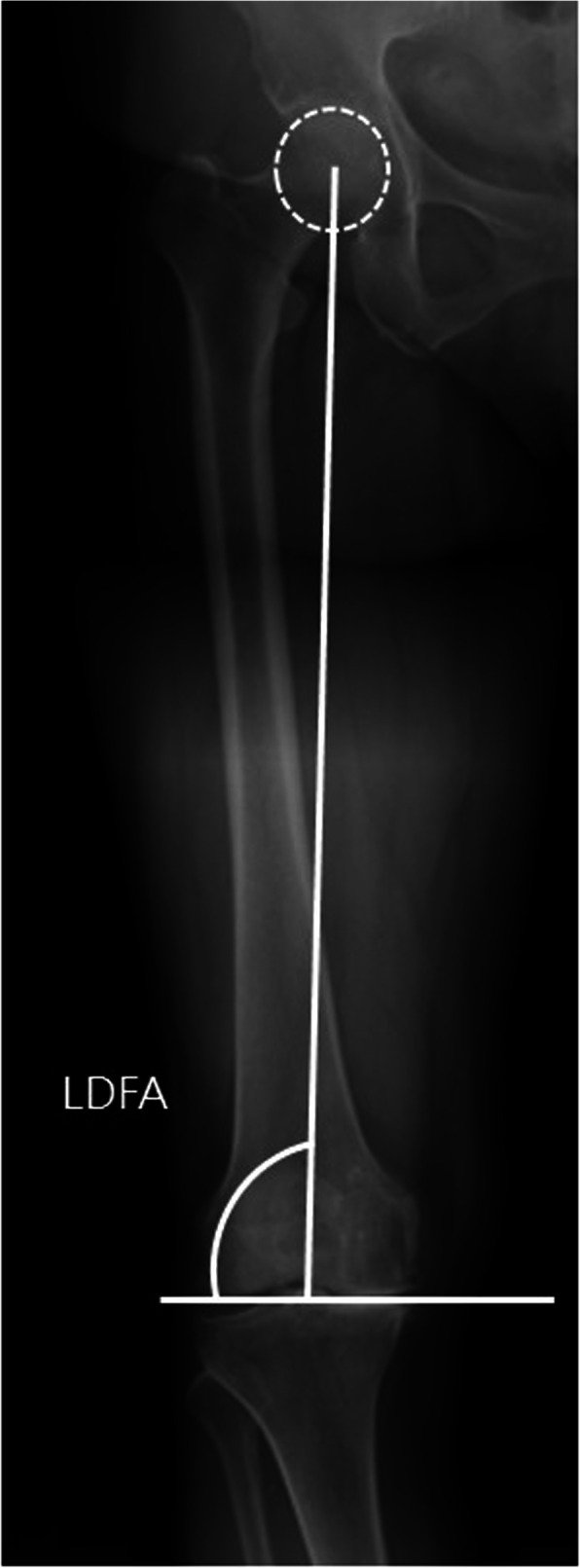
The lateral distal femur angle (LDFA) is measured on the whole-leg weight-bearing radiograph. It is defined as the lateral angle formed between the mechanical axis line of the femur (direct line from the center of femoral head to the top of the femoral notch) of the femur and knee joint line of the femur

The LDFA was defined as the lateral angle formed between the mechanical axis line of the femur and the knee joint line of the femur [19] (Fig. 2).

**Fig. 3:**
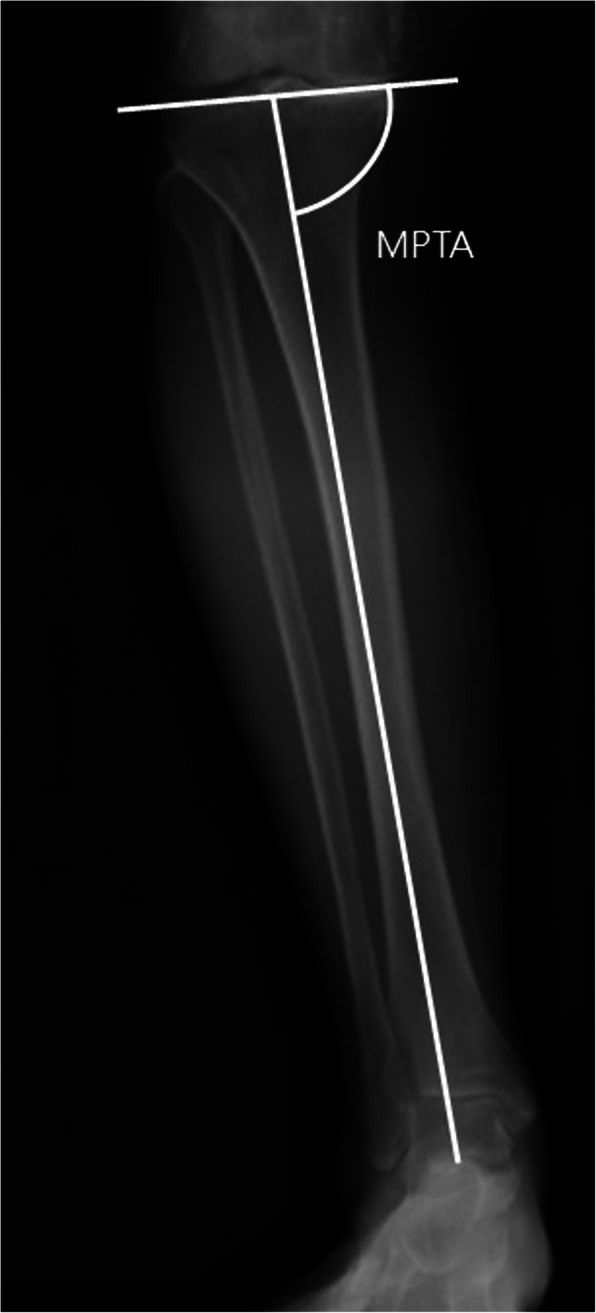
The medial tibial plateau angle (MTPA) is measured on the whole-leg weight-bearing radiograph. It is defined as the medial angle between the tibial articular marginal line and the mechanical axis line of the tibia (line from the ankle center to the center of the tibial spines)

The MTPA determines the inclination of the tibial plateau, which is defined as the medial angle between the mechanical axis line of the tibia (line from the ankle center to the center of the tibial spines) and the knee joint line of the tibia [19]. (Fig. 3).

The BMD was measured in the femoral neck through use of dual X-ray absorptiometry using GE apparatus. The BMI was calculated as the patient’s weight divided by height squared. An individual’s status of osteoporosis was defined as BMD T-score being ≤-2.5.

Image analysis was performed by two observers, one orthopedic surgeon (C.E Hsu; 14- years’ experience in arthroplastic surgery) and one resident (P.K. Wu 2-years’ experience in arthroplastic surgery) who were blinded to the BMD and of the patients.

The lower extremity varus malalignment was defined as the HKAA being < 175°. Varus inclination of the tibial plateau was defined as the MTPA being < 85°. Femur varus deformity was defined as the LDFA being > 85° [19]. The above described angles on a standardized whole leg weight bearing radiograph from the picture archiving and communication system (PACS) of our hospital. If the values did not match between the 2 observers, the 2 observers would then re-read the radiographs and determine the final values after reaching a consensus. 

### Statistical analysis

Data analysis was performed using SPSS software (Version 19.0; Chicago, Illinois). Univariate analysis was performed using frequencies for descriptive statistics. The Chi-square test was used in the analysis of categorical variables. Logistic regression was performed to calculate the crude odds ratio of each variable that contributed to the inclination of the tibial plateau. Multivariate logistic regression was performed to evaluate the adjusted odds ratio of variables with a *P* value ≤ 0.05. The kappa statistic (K) was done to estimate the proportion of agreement of both observers for the lower extremity varus malalignment, varus inclination of the tibial plateau and the femur varus deformity. The k values were interpreted as follows: k values between 0.00 and 0.20 represented poor; k values between 0.21 and 0.40 represented fair; k values between 0.41 and 0.60 represented moderate; k values between 0.61 and 0.80 represented good; k values between 0.81 and 1.00 represented excellent. Correlations were considered significant if *p* values were less than 0.05 (two-sided).

## Results

Table 1 summarizes the data of the 90 patients according to the presence of varus inclination of the tibial plateau (MTPA < 85°). This occurred in 32 (35.6 %) of the patients. Factors which had a significant association with varus inclination of the tibial plateau were age, lower extremity varus malalignment (HKAA < 175°) and osteoporosis (BMD T-score < -2.5) (*P* = 0.003, 0.001 and 0.011, respectively). Other variables, including Kellgren-Lawrence grade, operation side, BMI, LDFA, as well as spinal compression fracture and hip fracture history, showed no significant association with varus inclination of the tibial plateau. There was good inter-observer agreement of both observers for lower extremity malalignment (k = 0.61, *P* < 0.05), Varus inclination of tibial plateau (k = 0.66, *P* < 0.05) and varus deformity of distal femur (k = 0.62, *P* < 0.05).

**Table 1:** Basic characteristic of 90 postmenopausal-women with osteoarthritis of knee

Variables	Total (%) *N*=90	Inclination of Proximal Tibia		*P* Value
		No (MPTA≥85°) *N*=58	Yes (MPTA<85°) *N*=32	
Age, Years				
(Mean±SD)	73±6.6	72±6.7	76±5.7	**0.003**
Operation Side				
Right (%)	46 (51)	30 (52)	16 (50)	1.000
Left (%)	44 (49)	28 (48)	16(50)	
OA grade				
III(%)	53 (59)	38 (66)	15 (47)	0.117
IV(%)	37 (41)	20 (34)	17 (53)	
BMI, Kg/m^2^				
(Mean±SD)	27±3.6	27±3.4	27±4.1	0.829
HKAA				
≥175° (%)	20 (22)	19 (33)	1 (3)	**0.001**
<175° (%)	70 (78)	39 (67)	31 (97)	
LDFA				
≤85° (%)	87 (97)	56 (97)	31(97)	1.000
>85° (%)	3 (3)	2 (3)	1 (3)	
Spine CompressionFracture				
No (%)	81 (90)	55 (95)	26(81)	0.063
Yes (%)	9 (10)	3 (5)	6 (19)	
Hip Fracture				
No (%)	72 (80)	45 (78)	27(84)	0.585
Yes (%)	18 (20)	13 (22)	5 (16)	
BMD T-score				
>-2.5 (%)	77 (86)	54 (93)	23 (72)	0.011
≤-2.5 (%)	13 (14)	4 (7)	9 (28)	

Table 2 shows the results of the multivariant regression analysis. Age, lower extremity varus malalignment (HKAA < 175°) and osteoporosis were associated with an increased risk of varus inclination of the tibial plateau (*P* = 0.033, 0.013 and 0.015 respectively.

**Table 2:** Multivariate regression of risk factors of varus inclination of proximal tibia

Risk Factors	Odds Ratio (95 % C.I.)	*P* Value
Age, Years	1.10 (1.008–1.203)	**0.033**
Operation Side		
Right	1.00 (Ref.)	
Left	1.39(0.475–4.07)	0.547
OA stage		
III	1.00 (Ref.)	
IV	1.37(0.422–4.444)	0.601
BMI, Kg/m^2^	0.991 (0.857–1.147)	0.903
HKAA		
≥175°	1.00 (Ref.)	
<175°	20.78(1.874–229.9)	**0.013**
LDFA		
>85°	1.00 (Ref.)	
≤85°	0.25 (0.015–4.150)	0.335
Spinal Compression Fracture		
No	1.00 (Ref.)	
Yes	1.21 (0.213–6.868)	0.829
Hip Fracture		
No	1.00 (Ref.)	
Yes	0.574 (0.131–2.574)	0.460
FN BMD T-score		
>-2.5	1.00 (Ref.)	
≤-2.5	10.310 (1.573–67.592)	**0.015**

Further stratification of the data revealed that osteoporosis had significant association with varus inclination of the tibial plateau in patients with lower extremity varus malalignment (HKAA < 175°) (*P* < 0.001). However, no statistical significance for osteoporosis in patients with normal lower extremity alignment (HKAA ≥ 175°) was observed.

## Discussion

The aim of our study was to find risk factors for varus inclination of the tibial plateau in postmenopausal women with advanced varus OA knee. We discovered that age, lower extremity varus malalignment (HKAA < 175°) and osteoporosis (T-score ≤-2.5) were significant risk factors of varus inclination of the tibial plateau. Osteoporosis continued to significantly contribute to varus inclination of the tibial plateau in patients with lower extremity varus malalignment (*P* < 0.001). However, for patient with normal lower extremity alignment, osteoporosis had no significant association with varus inclination of the tibial plateau.

The first national Health and Nutrition Examination Survey (HANES I) that the incidence of osteoporosis and osteoarthritis of the knee both rapidly increased in postmenopausal women [9]. The relationship between osteoporosis and osteoarthritis of the knee (OA knee) has aroused interest in the medical community throughout many studies. Early studies focused on the association of BMD and knee joint space narrowing and found that BMD was not necessarily associated with joint space narrowing. [20–26]. Whether osteoporosis affects the progression of osteoarthritis of the knee remains controversial. However, the joint space narrowing may not be an ideal parameter to reflect the effect of osteoporosis on the OA knee progression because it can be affected by ligament laxity, meniscus injury, cartilage wearing and especially the weight bearing status [27–31]. The morphologic change of tibial plateau may be a better parameter which has less confounding factors to observe the effect of BMD on OA knee.

Insufficiency fracture at the medial compartment of the tibial plateau has been advocated as a cause for varus inclination of the tibial plateau [10, 11, 32]. Previous studies have also found that a more medial tibial condyle sclerosis is associated with a more severe varus OA knee or medial knee joint line narrowing [33–35]. The increased bone density at the medial compartment of the tibial plateau is similar to the finding of increased bone density along the compressed endplate in spinal compression fractures [36]. This change is usually chronic and progresses asymptomatically. However, when it reaches a certain threshold, it becomes symptomatic and precipitates the onset of OA knee [6]. Higano et al. found that the varus inclination of the tibial plateau increases in all non-OA, early OA and advanced OA knee patients over the course of a 21-year period follow-up. Patients with advanced OA knee experienced significantly more severe varus inclination of the tibial plateau than those with non-OA and early-OA knee [6]. Matsumoto et al. reported that the varus inclination of the tibial plateau maintained an approximately 86° angle in normal subjects, as well as those with early stage OA, but progressed to 84° in subjects with moderate or worse OA [3]. We believe that the increased varus inclination of the tibial plateau is caused by chronic trabecular microfractures at the medial compartment of the proximal tibia. In patients diagnosed with osteoporosis, this change is even more rapid and severe.

Two additional risk factors of varus inclination of the tibial plateau are age and lower extremity varus malalignment. Age is associated with both a lower BMD and a prolonged repetitive force on the medial compartment of the tibial plateau, which in turn may induce an insuficiency fracture. Our findings are in concordance with the findings of previous studies [3, 6, 37]. We also discovered that another significant risk factor was lower extremity varus malalignment. Lower extremity varus malalignment is related to an increased stress on the medial compartment of the tibia plateau, which yields cartilage wearing and progression of varus OA knee [2–5, 37–40]. Higano et al. reported that both lower extremity varus malalignment (HKAA < 175°) along with varus inclination of the tibial plateau (MTPA < 85°) could be observed in advanced OA patients while they were young and asymptomatic [6]. However, Cooke et al. reported that a significant difference in LDFA, but not MTPA, could be found between symptomatic varus OA knee patients and healthy subjects experiencing lower extremity varus malalignment [37]. Thus, Matsumoto et al. advocated that the progression of varus OA knee is initiated from lateral femoral bowing (increased LDFA), followed by medial joint space narrowing and finally tibial plateau compression [3]. As well as corresponding to previous studies [3, 37], our findings also elucidate that osteoporosis is an important risk factor for the progression when extremity varus malalignment coexists.

Two studies have been conducted surrounding the association of varus inclination of the tibial plateau and BMD [32, 41]. Terauchi et al. studied 37 patients and revealed a significant association between low BMD of the lumbar spine and varus inclination of the tibial plateau [32]. In contrast, Akamatsu et al. studied 135 postmenopausal women and reported that lumbar spine BMD has no association with varus inclination of the tibial plateau. Additionally, the team reported a negative correlation between varus inclination of the tibial plateau and BMD at the ipsilateral femoral neck [41]. In our current study, we studied 90 postmenopausal women and used ipsilateral femoral BMD as a predictor for varus inclination of the tibial plateau but not spinal BMD, due to degenerative spinal diseases tending to be associated with increased lumbar spine BMD, which may be a strong confounding factor for our observation [42]. Our finding was that patients with varus inclination of tibial plateau had significant higher incidence of low femoral neck BMD which is in accordance to the findings of Akamatsu et al. As opposed to previous studies which used an non-weight bearing anteroposterior radiograph of the knee to measure parameters, including the femoral condylar-shaft angle, tibial plateau-shaft angle, and femoral tibial angle [32, 41], our parameters were measured from weight-bearing, entire leg standing radiograph, which is reportedly more accurate and reliable [31, 43, 44]. In additional to reconfirming the finding of Akamatsu et al., our findings also elucidate that lower extremity varus malalignment is an important risk factor for varus inclination of tibial plateau when osteoporosis coexists.

Osteoporosis is also reportedly associated with subchondral insufficiency fracture of the knee (SIFK) (formerly known as spontaneous osteonecrosis of the knee or SONK) [45]. The decreased BMD maybe an aggravation factor in the progression of SIFK when the posterior medial meniscus root is disrupted and tibiofemoral contact pressures is increased. Though SIFK occurs more commonly in the weight bearing femoral condyle, it can occur in the tibial plateau [46]. The association of SIFK and varus inclination of tibial plateau is still unclear and should be elucidated in future studies.

This study has several limitations. First, the design was cross-sectional rather than longitudinal. The causality of malalignment, varus inclination of the tibial plateau and osteoporosis cannot be determined. Although our findings suggest the possible reason of for varus inclination of the tibial plateau, a prospective longitudinal study should be conducted in order to verify the results of present study. Second, the sample size was relatively small. Factors that may confound the effects of osteoporosis on varus inclination of the tibial plateau, such as lifestyle and bone quality, serum level of vitamin D, were not included in our analysis.

## Conclusions

In conclusion, we examined 90 postmenopausal women with OA knee and found that age, lower extremity malalignment and osteoporosis are major risk factors of varus inclination of the tibial plateau. More attention needs to be paid on the progression of varus OA knee in postmenopausal women who are experiencing osteoporosis and lower extremity varus malalignment simultaneously.

## Data Availability

The datasets used and analyzed during the current study are available from the corresponding author on reasonable request.

## Abbreviations

OA: Osteoarthritis
MTPA: Medial Tibial Plateau Angle
BMD: Bone Mineral Density
BMI: Body Mass Index
LDFA: Lateral Distal Femur Angle
HKAA: Hip-Knee-Ankle Angle
SIFK: Subchondral Insufficiency Fracture of the Knee
